# Supplementary material for: Evolution of Cherries (Prunus Subgenus Cerasus) Based on Chloroplast Genomes
Source: Int J Mol Sci. 2023 Oct 26;24(21):15612. doi: 10.3390/ijms242115612 (PMC10650623; doi:10.3390/ijms242115612)
Supplement: Supplementary file 1 [file ijms-24-15612-s001.zip › Figure S1.pdf]

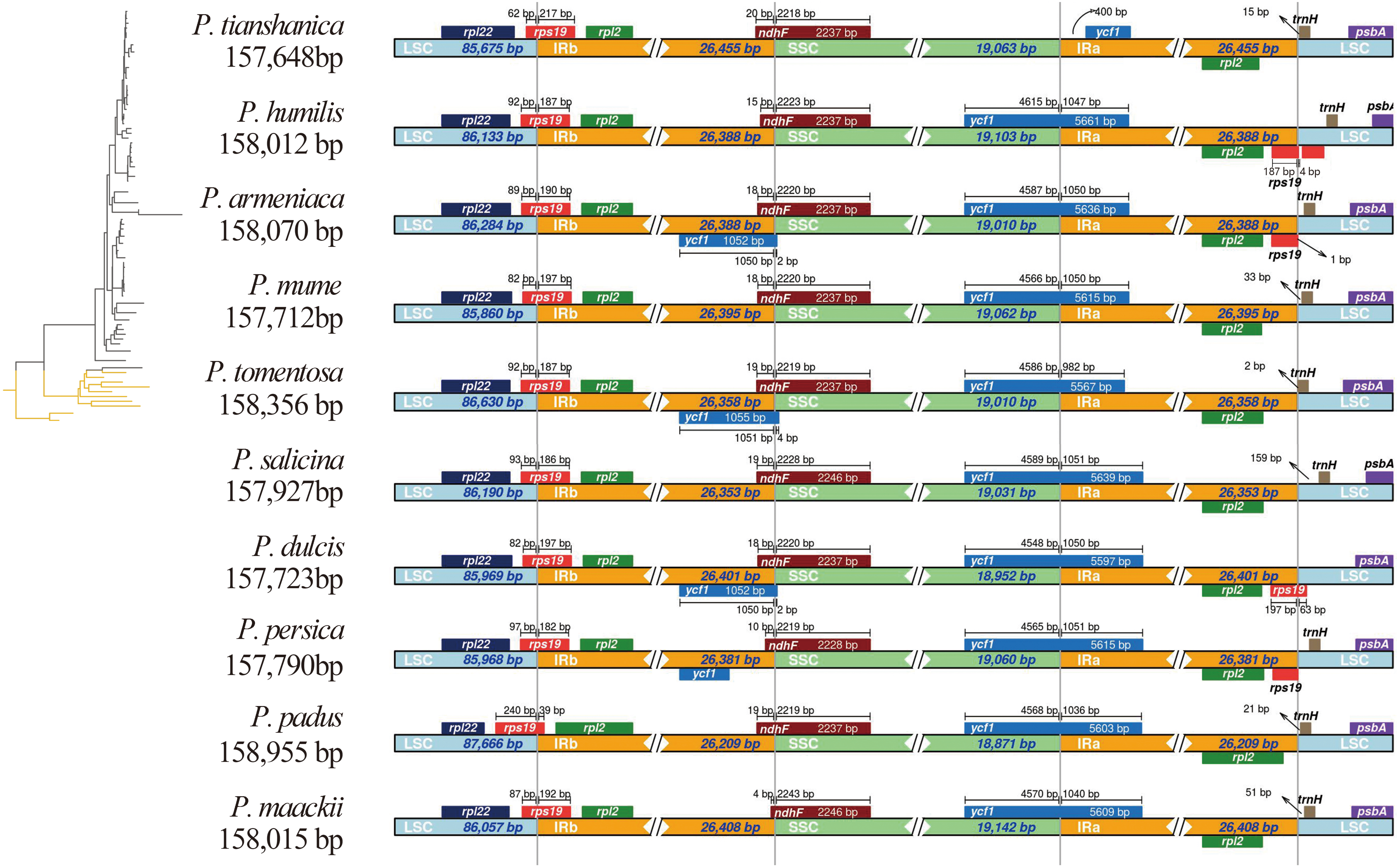
P. tianshanica 157,648bp
P. humilis 158,012 bp
P. armeniaca 158,070 bp
P. mume 157,712bp
P. tomentosa 158,356 bp
P. salicina 157,927bp
P. dulcis 157,723bp
P. persica 157,790bp
P. padus 158,955 bp
P. maackii 158,015 bp
LSC
IRb
SSC
IRa
rpl22
rps19
rpl2
ndhF
ycf1
trnH
psbA

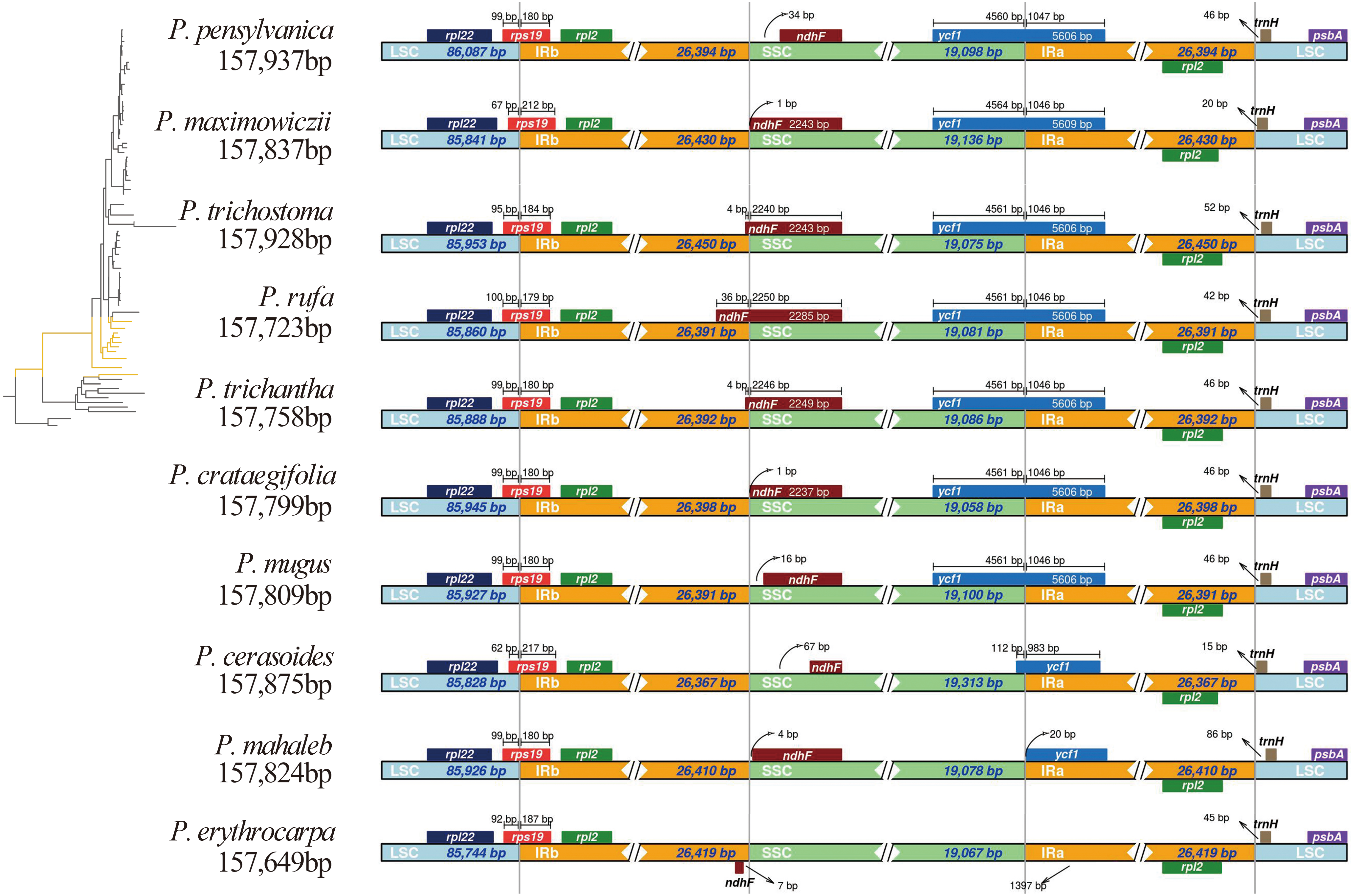

P. pensylvanica
157,937bp
99 bp 180 bp
34 bp
4560 bp 1047 bp
46 bp
rpl22 rps19 rpl2 ndhF ycf1 5606 bp trnH psbA
LSC 86,087 bp IRb 26,394 bp SSC 19,098 bp IRa 26,394 bp LSC
rpl2
P. maximowiczii
157,837bp
67 bp 212 bp
1 bp
4564 bp 1046 bp
20 bp
rpl22 rps19 rpl2 ndhF 2243 bp ycf1 5609 bp trnH psbA
LSC 85,841 bp IRb 26,430 bp SSC 19,136 bp IRa 26,430 bp LSC
rpl2
P. trichostoma
157,928bp
95 bp 184 bp
4 bp 2240 bp
4561 bp 1046 bp
52 bp
rpl22 rps19 rpl2 ndhF 2243 bp ycf1 5606 bp trnH psbA
LSC 85,953 bp IRb 26,450 bp SSC 19,075 bp IRa 26,450 bp LSC
rpl2
P. rufa
157,723bp
100 bp 179 bp
36 bp 2250 bp
4561 bp 1046 bp
42 bp
rpl22 rps19 rpl2 ndhF 2285 bp ycf1 5606 bp trnH psbA
LSC 85,860 bp IRb 26,391 bp SSC 19,081 bp IRa 26,391 bp LSC
rpl2
P. trichantha
157,758bp
99 bp 180 bp
4 bp 2246 bp
4561 bp 1046 bp
46 bp
rpl22 rps19 rpl2 ndhF 2249 bp ycf1 5606 bp trnH psbA
LSC 85,888 bp IRb 26,392 bp SSC 19,086 bp IRa 26,392 bp LSC
rpl2
P. crataegifolia
157,799bp
99 bp 180 bp
1 bp
4561 bp 1046 bp
46 bp
rpl22 rps19 rpl2 ndhF 2237 bp ycf1 5606 bp trnH psbA
LSC 85,945 bp IRb 26,398 bp SSC 19,058 bp IRa 26,398 bp LSC
rpl2
P. mugus
157,809bp
99 bp 180 bp
16 bp
4561 bp 1046 bp
46 bp
rpl22 rps19 rpl2 ndhF ycf1 5606 bp trnH psbA
LSC 85,927 bp IRb 26,391 bp SSC 19,100 bp IRa 26,391 bp LSC
rpl2
P. cerasoides
157,875bp
62 bp 217 bp
67 bp
112 bp 983 bp
15 bp
rpl22 rps19 rpl2 ndhF ycf1 trnH psbA
LSC 85,828 bp IRb 26,367 bp SSC 19,313 bp IRa 26,367 bp LSC
rpl2
P. mahaleb
157,824bp
99 bp 180 bp
4 bp
20 bp
86 bp
rpl22 rps19 rpl2 ndhF ycf1 trnH psbA
LSC 85,926 bp IRb 26,410 bp SSC 19,078 bp IRa 26,410 bp LSC
rpl2
P. erythrocarpa
157,649bp
92 bp 187 bp
45 bp
rpl22 rps19 rpl2 trnH psbA
LSC 85,744 bp IRb 26,419 bp SSC 19,067 bp IRa 26,419 bp LSC
rpl2
ndhF 7 bp
1397 bp

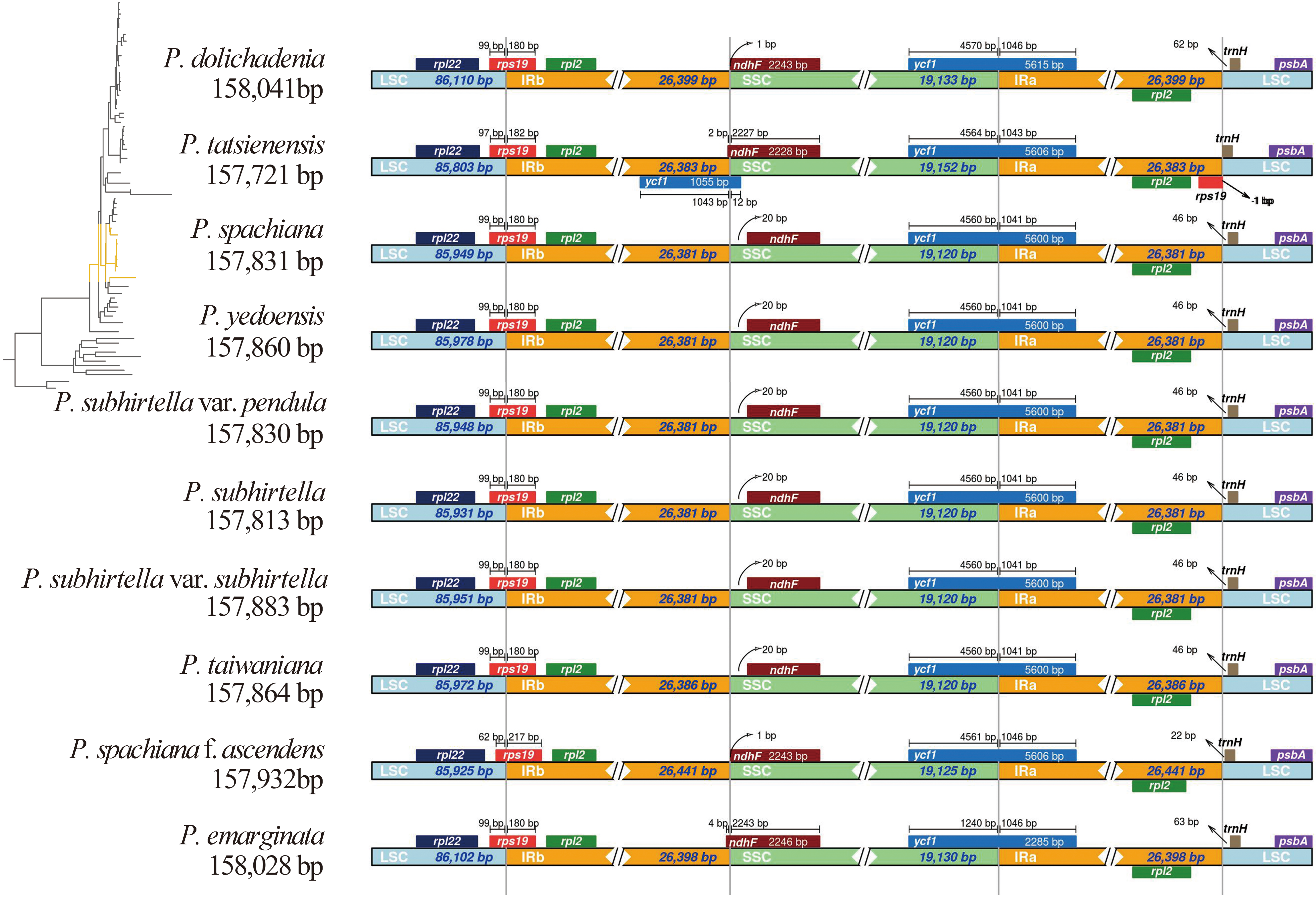

P. dolichadenia
158,041bp
99 bp 180 bp
rpl22
rps19
rpl2
1 bp
ndhF 2243 bp
4570 bp 1046 bp
ycf1 5615 bp
62 bp
trnH
psbA
LSC 86,110 bp IRb
26,399 bp SSC
19,133 bp IRa
26,399 bp LSC
P. tatsienensis
157,721 bp
97 bp 182 bp
2 bp 2227 bp
ndhF 2228 bp
4564 bp 1043 bp
ycf1 5606 bp
LSC 85,803 bp IRb
26,383 bp SSC
19,152 bp IRa
26,383 bp LSC
ycf1 1055 bp
1043 bp 12 bp
rps19
-1 bp
P. spachiana
157,831 bp
99 bp 180 bp
20 bp
ndhF
4560 bp 1041 bp
ycf1 5600 bp
46 bp
LSC 85,949 bp IRb
26,381 bp SSC
19,120 bp IRa
26,381 bp LSC
P. yedoensis
157,860 bp
LSC 85,978 bp IRb
26,381 bp SSC
19,120 bp IRa
26,381 bp LSC
P. subhirtella var. pendula
157,830 bp
LSC 85,948 bp IRb
26,381 bp SSC
19,120 bp IRa
26,381 bp LSC
P. subhirtella
157,813 bp
LSC 85,931 bp IRb
26,381 bp SSC
19,120 bp IRa
26,381 bp LSC
P. subhirtella var. subhirtella
157,883 bp
LSC 85,951 bp IRb
26,381 bp SSC
19,120 bp IRa
26,381 bp LSC
P. taiwaniana
157,864 bp
LSC 85,972 bp IRb
26,386 bp SSC
19,120 bp IRa
26,386 bp LSC
P. spachiana f. ascendens
157,932bp
62 bp 217 bp
1 bp
ndhF 2243 bp
4561 bp 1046 bp
ycf1 5606 bp
22 bp
LSC 85,925 bp IRb
26,441 bp SSC
19,125 bp IRa
26,441 bp LSC
P. emarginata
158,028 bp
99 bp 180 bp
4 bp 2243 bp
ndhF 2246 bp
1240 bp 1046 bp
ycf1 2285 bp
63 bp
LSC 86,102 bp IRb
26,398 bp SSC
19,130 bp IRa
26,398 bp LSC

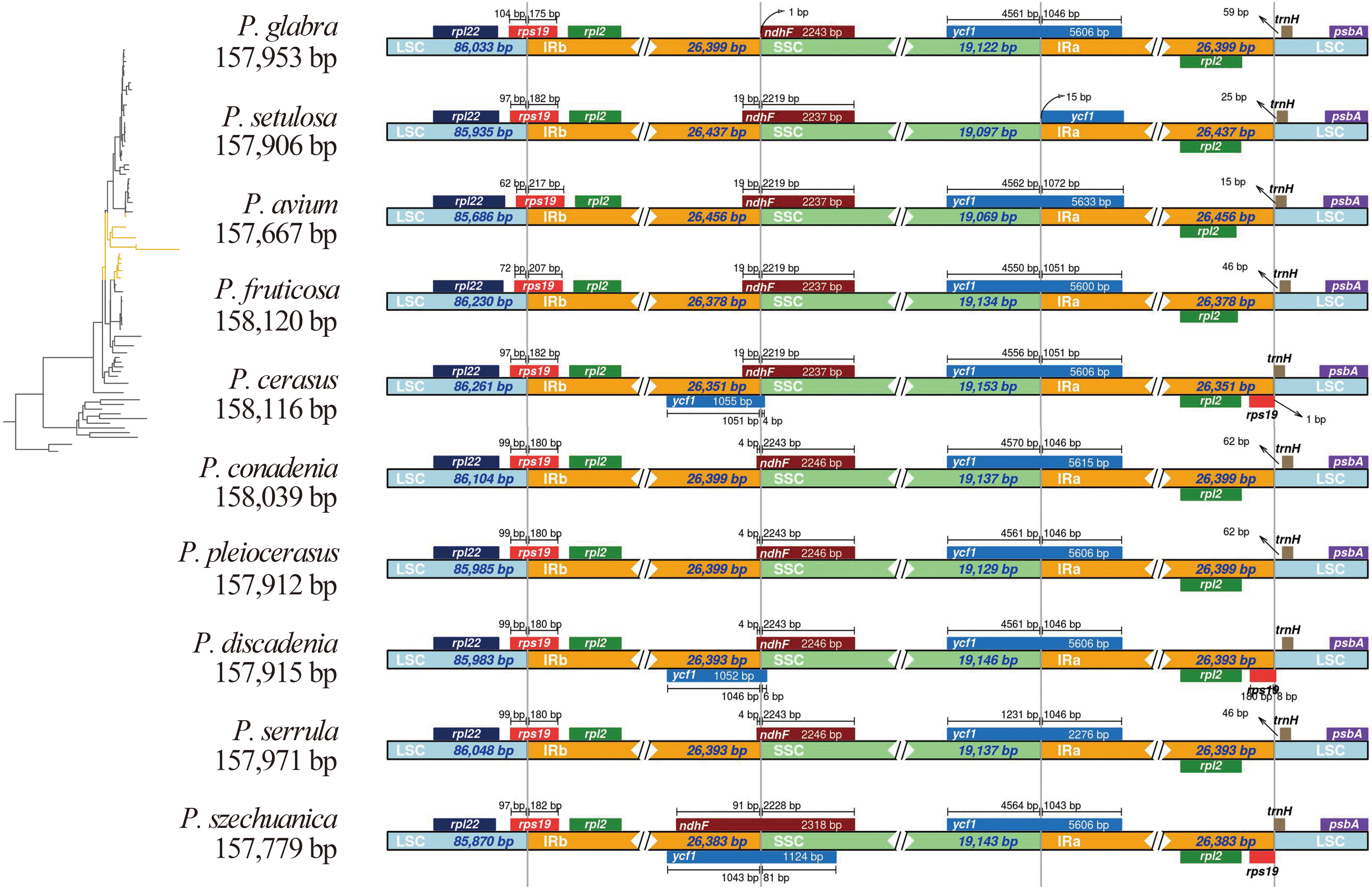

P. glabra
157,953 bp
LSC 86,033 bp
IRb 26,399 bp
SSC 19,122 bp
IRa 26,399 bp
P. setulosa
157,906 bp
LSC 85,935 bp
IRb 26,437 bp
SSC 19,097 bp
IRa 26,437 bp
P. avium
157,667 bp
LSC 85,686 bp
IRb 26,456 bp
SSC 19,069 bp
IRa 26,456 bp
P. fruticosa
158,120 bp
LSC 86,230 bp
IRb 26,378 bp
SSC 19,134 bp
IRa 26,378 bp
P. cerasus
158,116 bp
LSC 86,261 bp
IRb 26,351 bp
SSC 19,153 bp
IRa 26,351 bp
P. conadenia
158,039 bp
LSC 86,104 bp
IRb 26,399 bp
SSC 19,137 bp
IRa 26,399 bp
P. pleiocerasus
157,912 bp
LSC 85,985 bp
IRb 26,399 bp
SSC 19,129 bp
IRa 26,399 bp
P. discadenia
157,915 bp
LSC 85,983 bp
IRb 26,393 bp
SSC 19,146 bp
IRa 26,393 bp
P. serrula
157,971 bp
LSC 86,048 bp
IRb 26,393 bp
SSC 19,137 bp
IRa 26,393 bp
P. szechuanica
157,779 bp
LSC 85,870 bp
IRb 26,383 bp
SSC 19,143 bp
IRa 26,383 bp
rpl22
rps19
rpl2
ndhF
ycf1
trnH
psbA

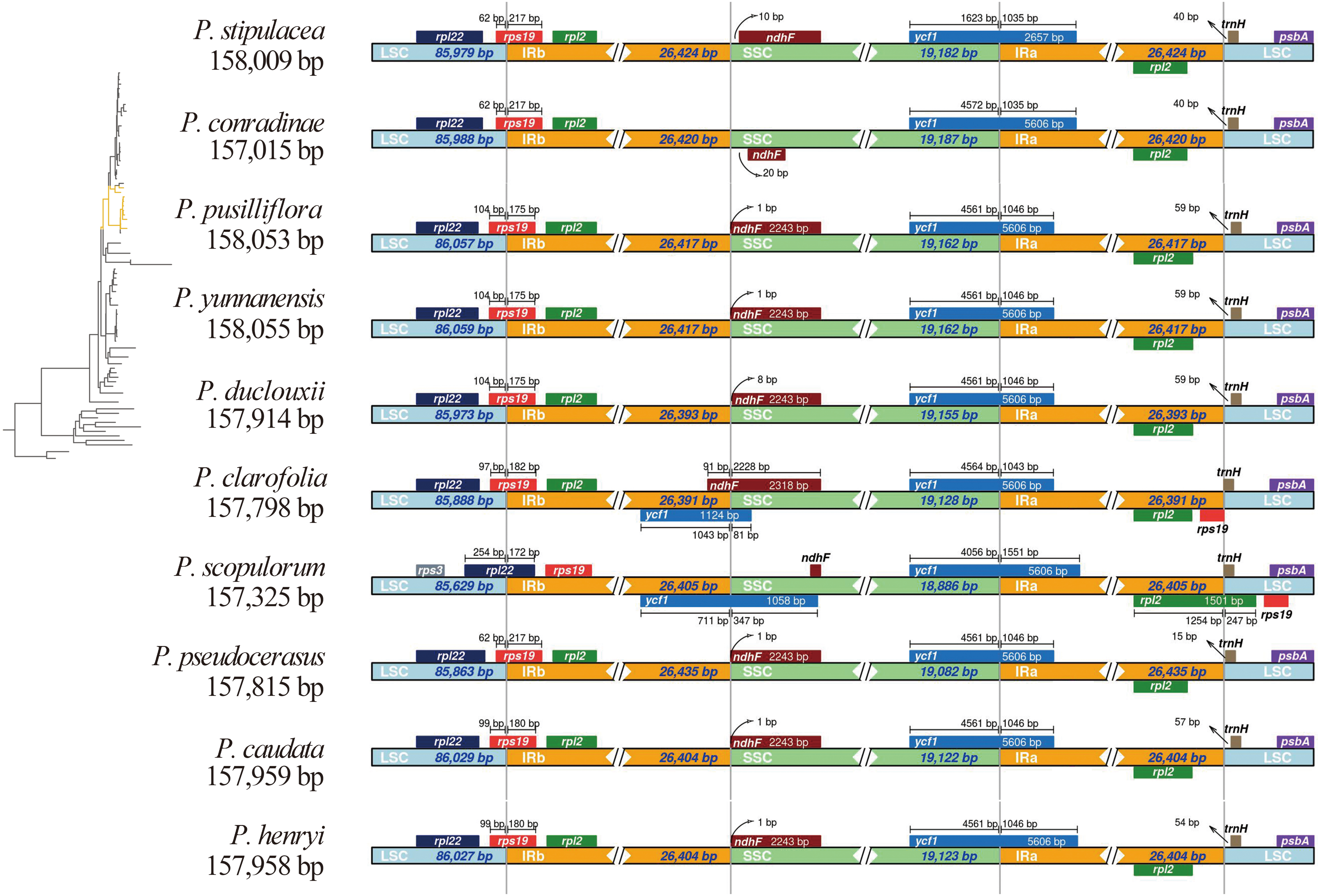

P. stipulacea
158,009 bp
62 bp 217 bp
rpl22 rps19 rpl2
10 bp
ndhF
1623 bp 1035 bp
ycf1 2657 bp
40 bp trnH psbA
LSC 85,979 bp IRb 26,424 bp SSC 19,182 bp IRa 26,424 bp LSC
rpl2
P. conradinae
157,015 bp
62 bp 217 bp
rpl22 rps19 rpl2
4572 bp 1035 bp
ycf1 5606 bp
40 bp trnH psbA
LSC 85,988 bp IRb 26,420 bp SSC 19,187 bp IRa 26,420 bp LSC
ndhF
20 bp
rpl2
P. pusilliflora
158,053 bp
104 bp 175 bp
rpl22 rps19 rpl2
1 bp
ndhF 2243 bp
4561 bp 1046 bp
ycf1 5606 bp
59 bp trnH psbA
LSC 86,057 bp IRb 26,417 bp SSC 19,162 bp IRa 26,417 bp LSC
rpl2
P. yunnanensis
158,055 bp
104 bp 175 bp
rpl22 rps19 rpl2
1 bp
ndhF 2243 bp
4561 bp 1046 bp
ycf1 5606 bp
59 bp trnH psbA
LSC 86,059 bp IRb 26,417 bp SSC 19,162 bp IRa 26,417 bp LSC
rpl2
P. duclouxii
157,914 bp
104 bp 175 bp
rpl22 rps19 rpl2
8 bp
ndhF 2243 bp
4561 bp 1046 bp
ycf1 5606 bp
59 bp trnH psbA
LSC 85,973 bp IRb 26,393 bp SSC 19,155 bp IRa 26,393 bp LSC
rpl2
P. clarofolia
157,798 bp
97 bp 182 bp
rpl22 rps19 rpl2
91 bp 2228 bp
ndhF 2318 bp
4564 bp 1043 bp
ycf1 5606 bp
trnH psbA
LSC 85,888 bp IRb 26,391 bp SSC 19,128 bp IRa 26,391 bp LSC
ycf1 1124 bp
1043 bp 81 bp
rpl2 rps19
P. scopulorum
157,325 bp
254 bp 172 bp
rps3 rpl22 rps19
ndhF
4056 bp 1551 bp
ycf1 5606 bp
trnH psbA
LSC 85,629 bp IRb 26,405 bp SSC 18,886 bp IRa 26,405 bp LSC
ycf1 1058 bp
711 bp 347 bp
rpl2 1501 bp
1254 bp 247 bp rps19
P. pseudocerasus
157,815 bp
62 bp 217 bp
rpl22 rps19 rpl2
1 bp
ndhF 2243 bp
4561 bp 1046 bp
ycf1 5606 bp
15 bp trnH psbA
LSC 85,863 bp IRb 26,435 bp SSC 19,082 bp IRa 26,435 bp LSC
rpl2
P. caudata
157,959 bp
99 bp 180 bp
rpl22 rps19 rpl2
1 bp
ndhF 2243 bp
4561 bp 1046 bp
ycf1 5606 bp
57 bp trnH psbA
LSC 86,029 bp IRb 26,404 bp SSC 19,122 bp IRa 26,404 bp LSC
rpl2
P. henryi
157,958 bp
99 bp 180 bp
rpl22 rps19 rpl2
1 bp
ndhF 2243 bp
4561 bp 1046 bp
ycf1 5606 bp
54 bp trnH psbA
LSC 86,027 bp IRb 26,404 bp SSC 19,123 bp IRa 26,404 bp LSC
rpl2

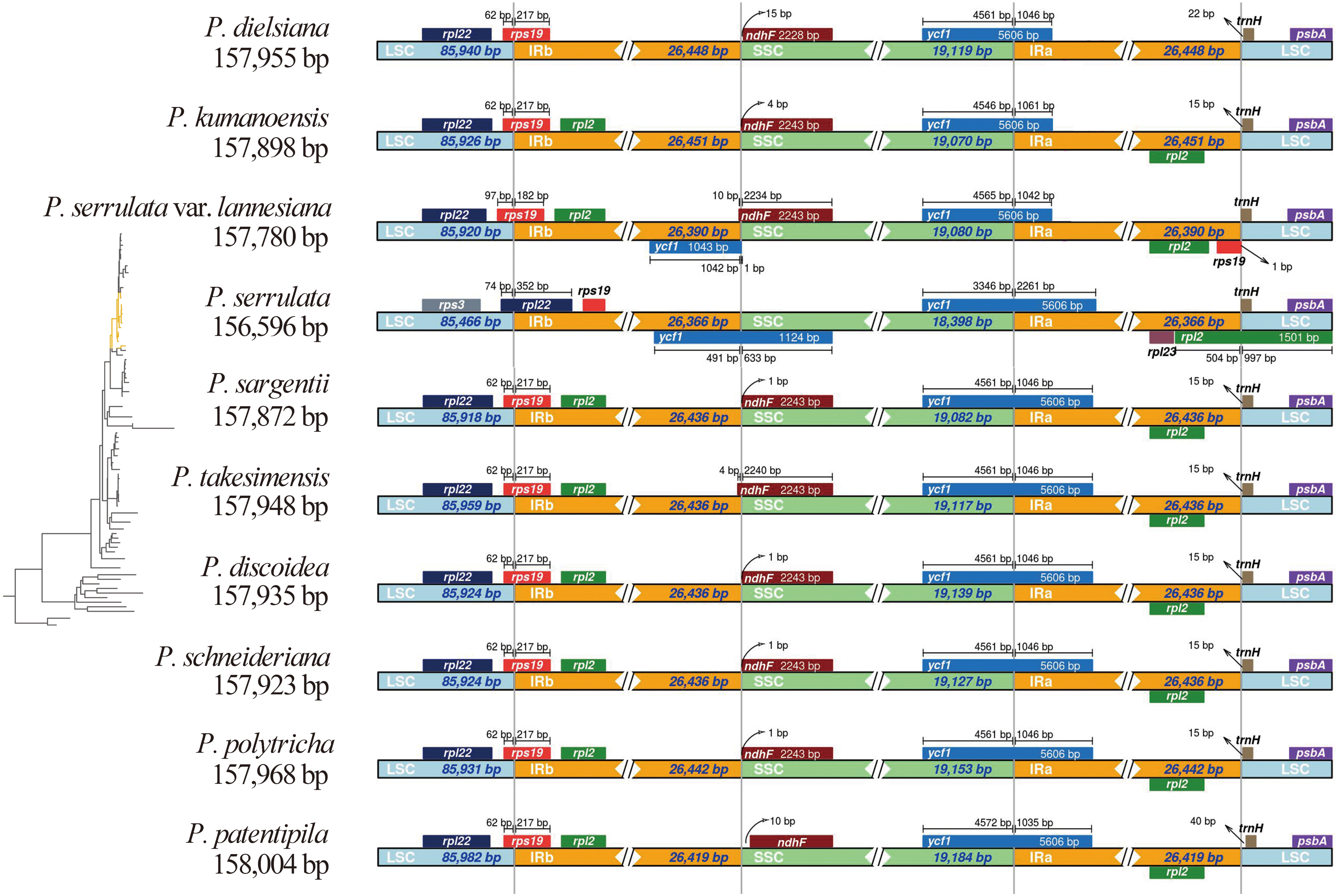

P. dielsiana
157,955 bp
P. kumanoensis
157,898 bp
P. serrulata var. lannesiana
157,780 bp
P. serrulata
156,596 bp
P. sargentii
157,872 bp
P. takesimensis
157,948 bp
P. discoidea
157,935 bp
P. schneideriana
157,923 bp
P. polytricha
157,968 bp
P. patentipila
158,004 bp
LSC
IRb
SSC
IRa
rpl22
rps19
rpl2
rps3
ndhF
ycf1
trnH
psbA
rpl23

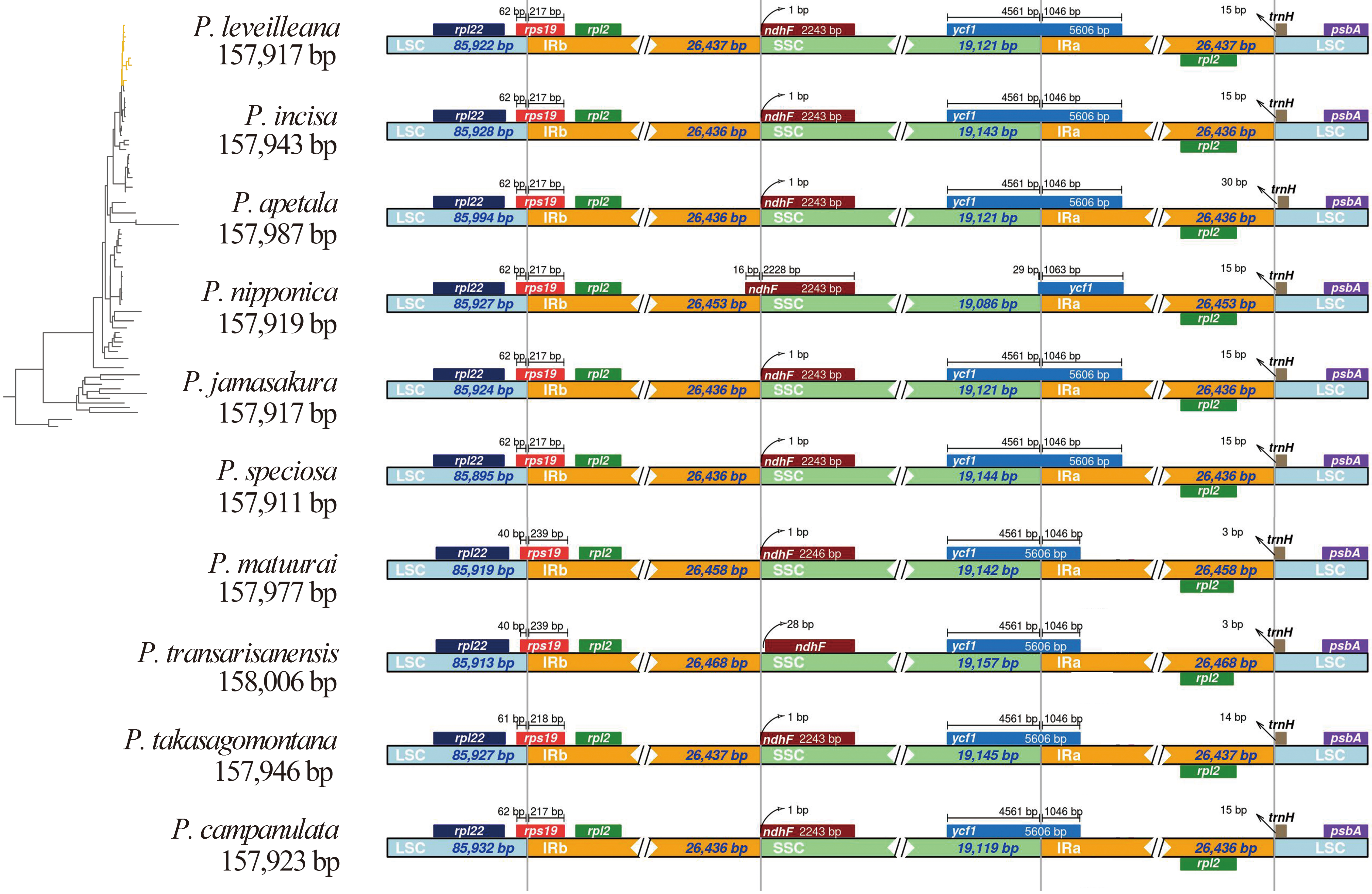

P. leveilleana
157,917 bp
85,922 bp
26,437 bp
19,121 bp
26,437 bp
P. incisa
157,943 bp
85,928 bp
26,436 bp
19,143 bp
26,436 bp
P. apetala
157,987 bp
85,994 bp
26,436 bp
19,121 bp
26,436 bp
P. nipponica
157,919 bp
85,927 bp
26,453 bp
19,086 bp
26,453 bp
P. jamasakura
157,917 bp
85,924 bp
26,436 bp
19,121 bp
26,436 bp
P. speciosa
157,911 bp
85,895 bp
26,436 bp
19,144 bp
26,436 bp
P. matuurai
157,977 bp
85,919 bp
26,458 bp
19,142 bp
26,458 bp
P. transarisanensis
158,006 bp
85,913 bp
26,468 bp
19,157 bp
26,468 bp
P. takasagomontana
157,946 bp
85,927 bp
26,437 bp
19,145 bp
26,437 bp
P. campanulata
157,923 bp
85,932 bp
26,436 bp
19,119 bp
26,436 bp
ndhF
ycf1
trnH
psbA
rpl2
