# Supplementary figures and images for: Evolution of Cherries (Prunus Subgenus Cerasus) Based on Chloroplast Genomes
Source: Int J Mol Sci. 2023 Oct 26;24(21):15612. doi: 10.3390/ijms242115612 (PMC10650623; doi:10.3390/ijms242115612)

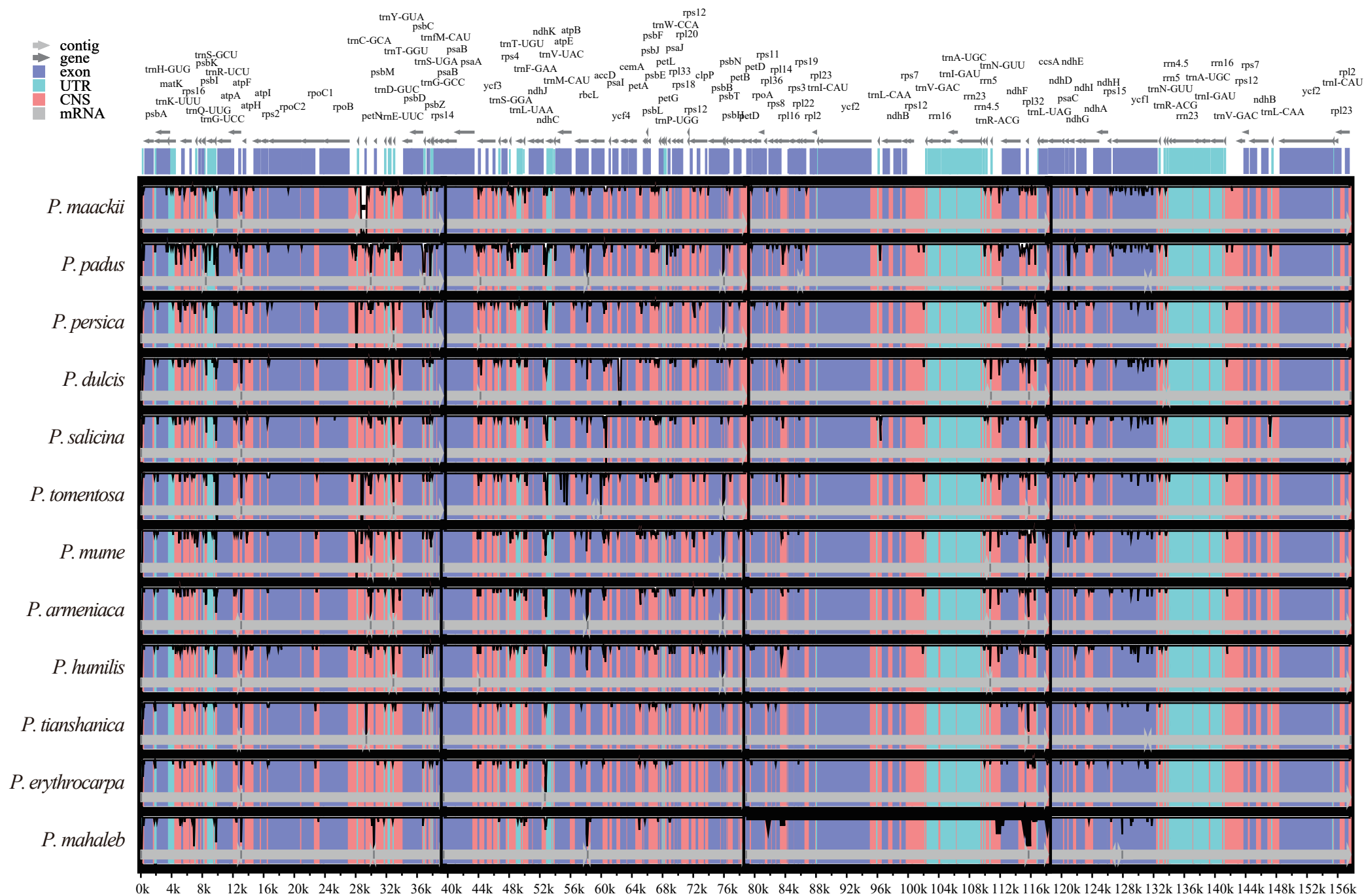

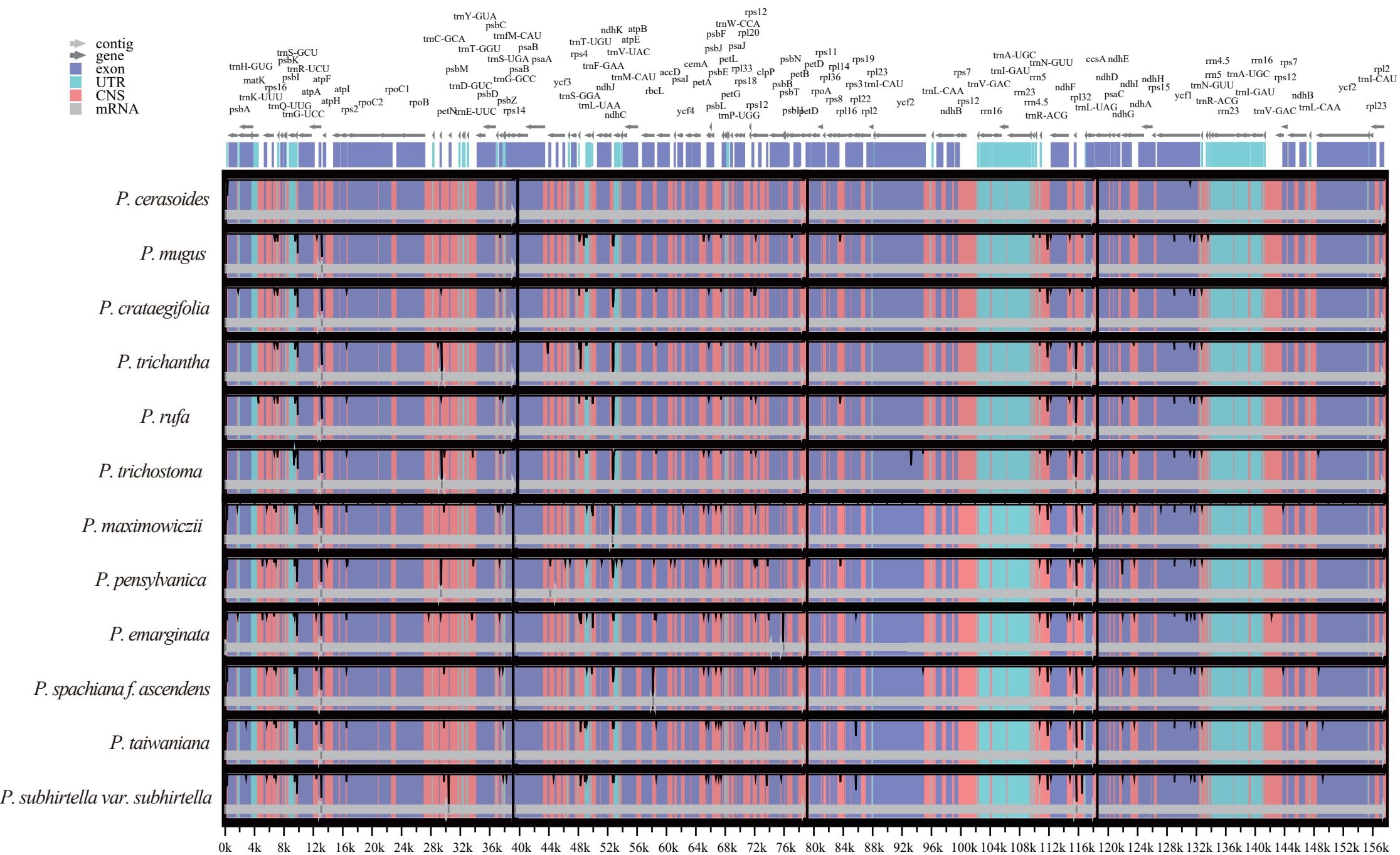

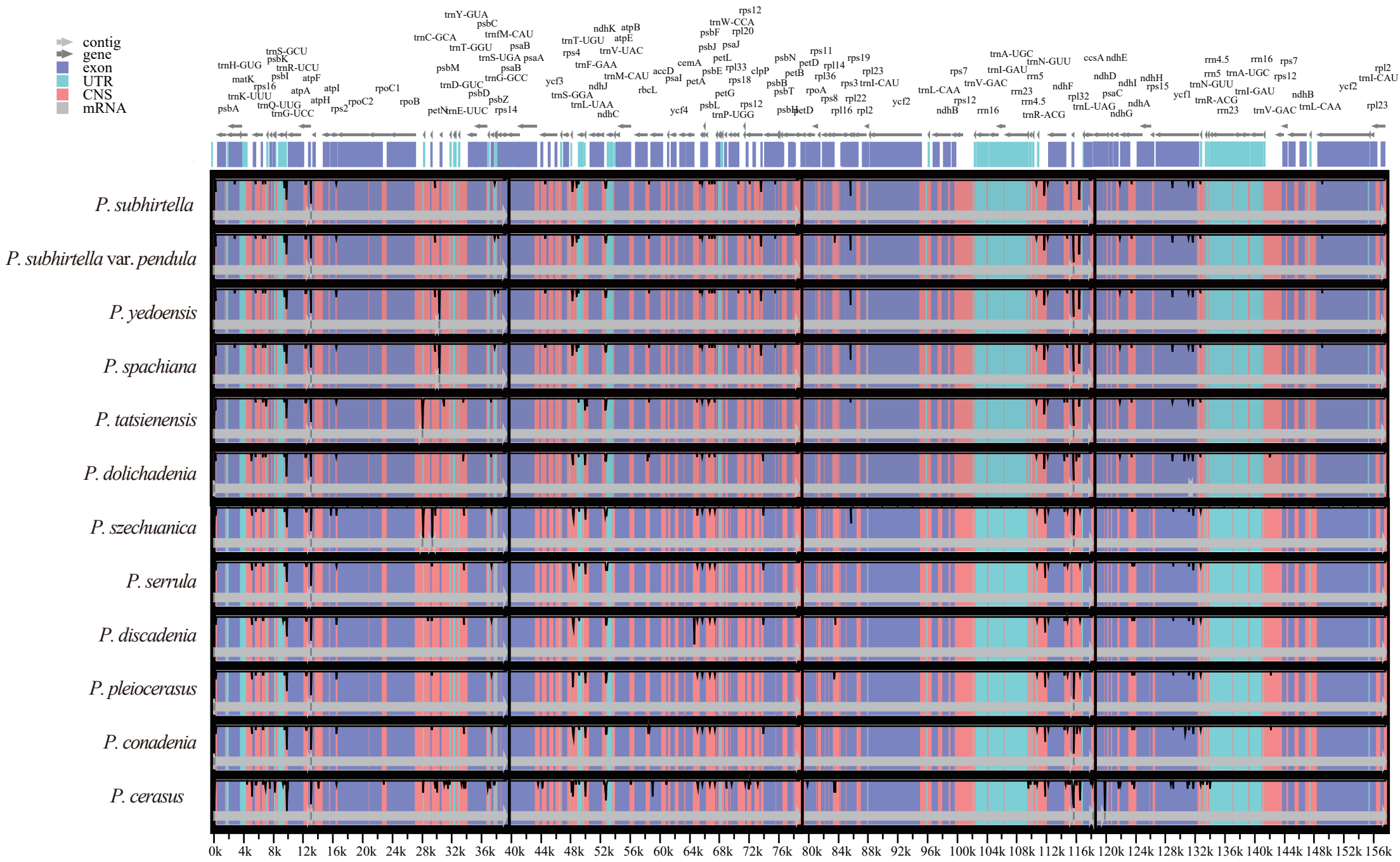

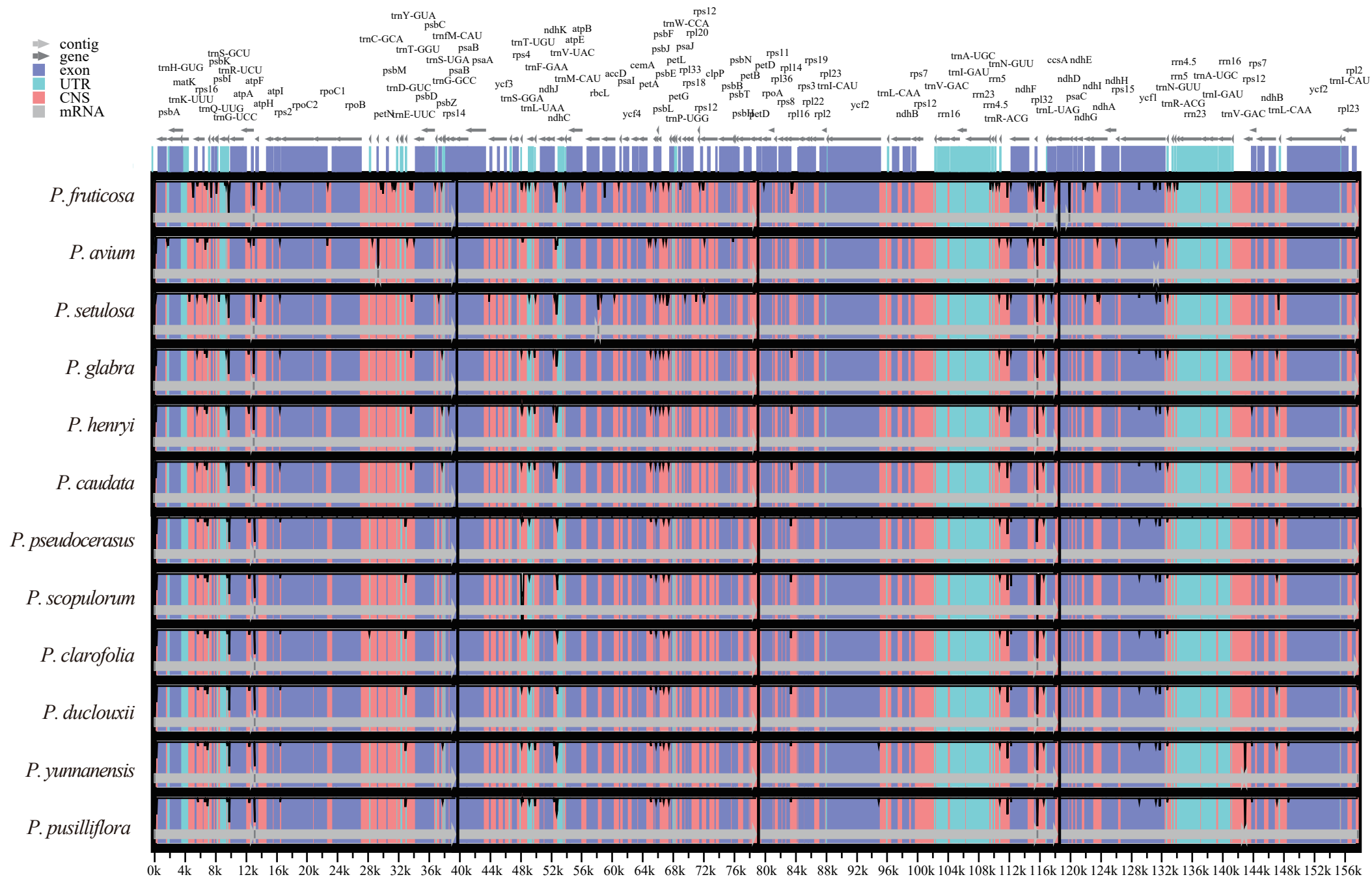

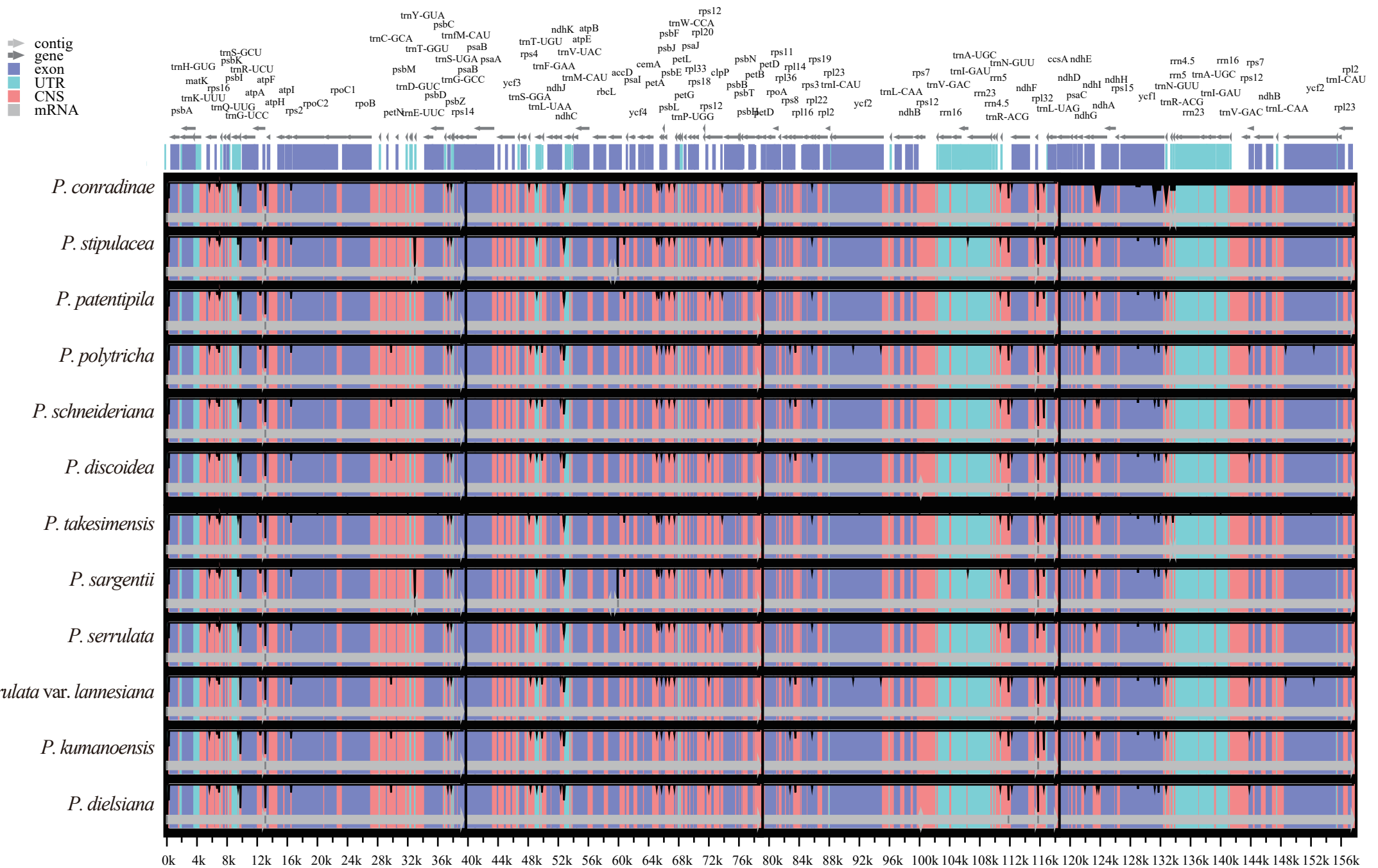

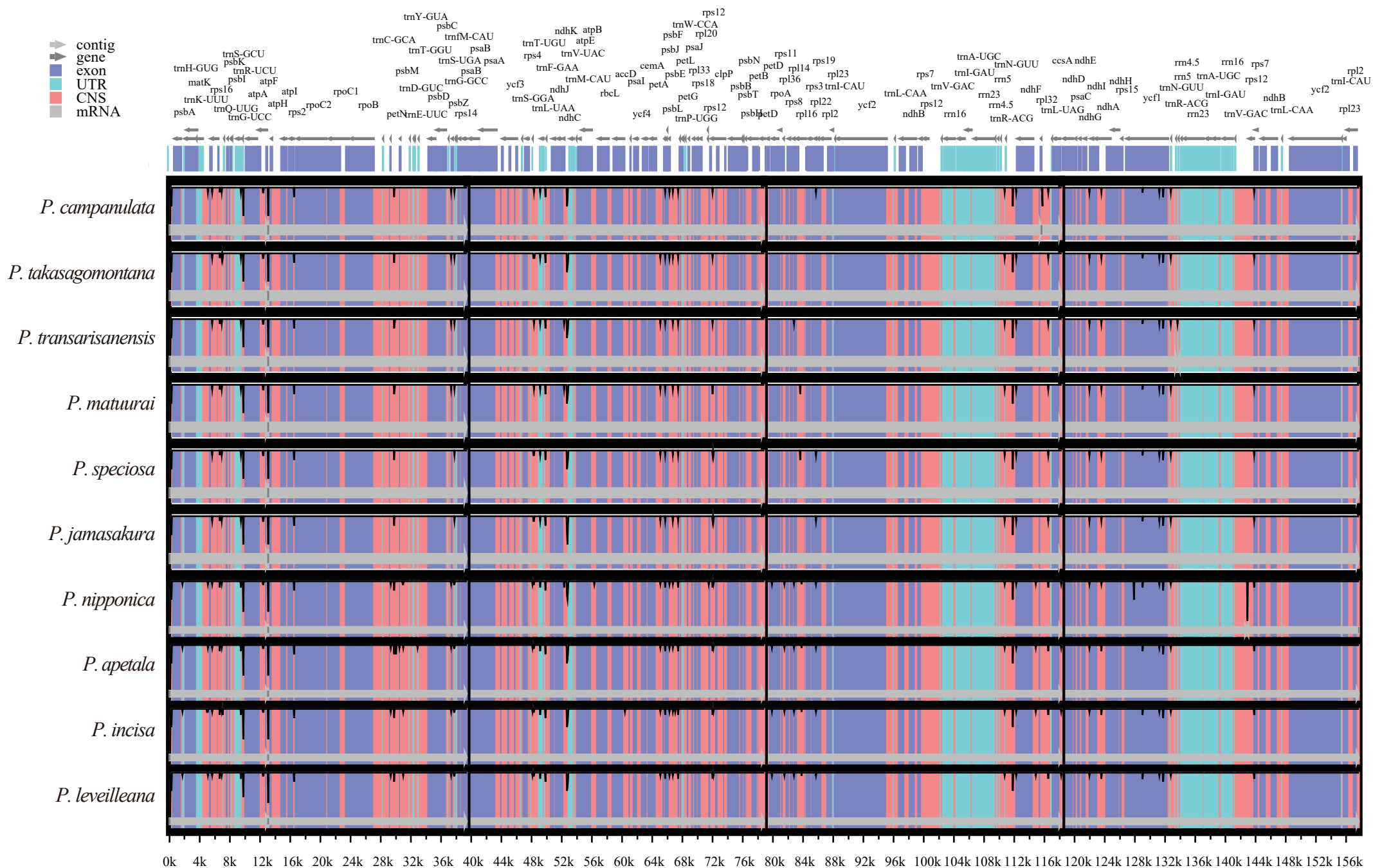

Supplement: Supplementary file 1 [file ijms-24-15612-s001.zip › Figure S2.pdf]

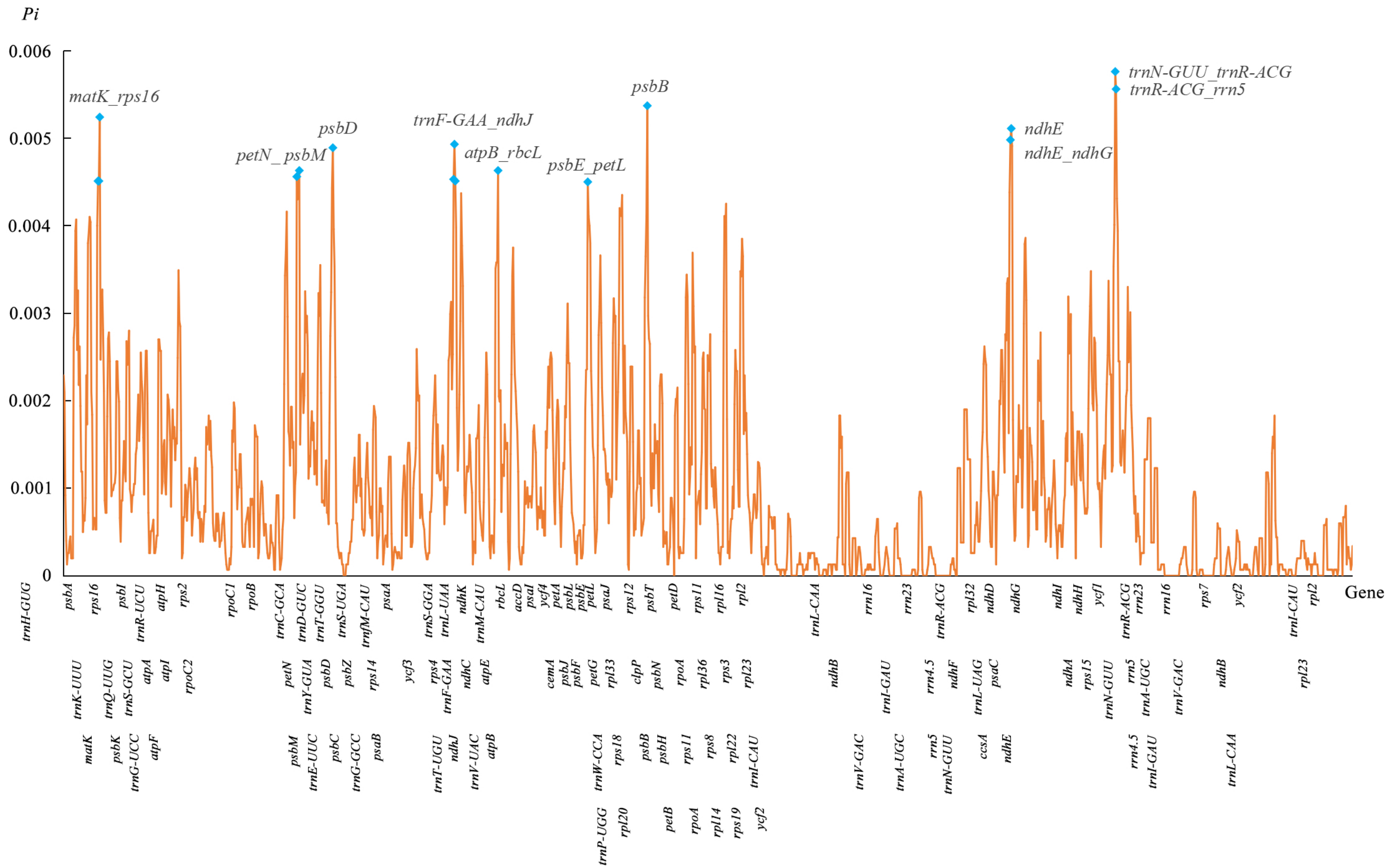

Supplement: Supplementary file 1 [file ijms-24-15612-s001.zip › Figure S3.pdf]

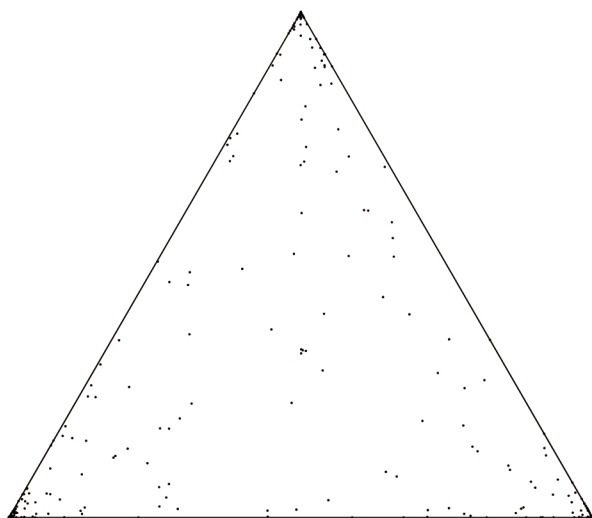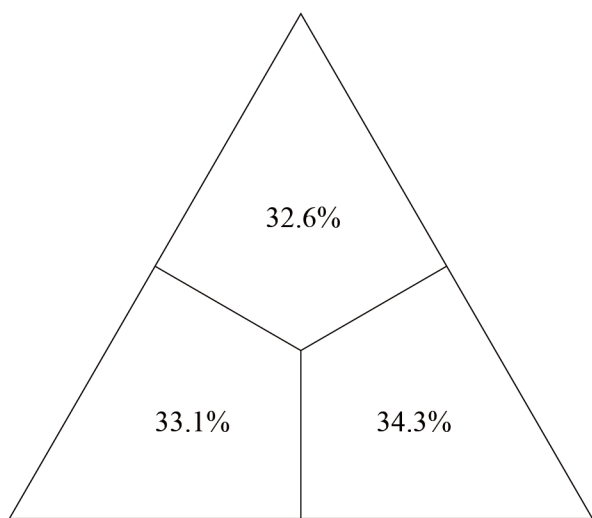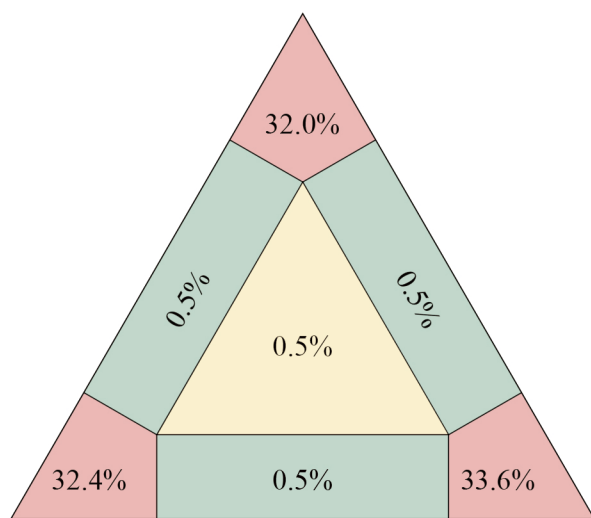

Tree-like  Net-like  Star-like

Supplement: Supplementary file 1 [file ijms-24-15612-s001.zip › Figure S4.pdf]

(a) (b)

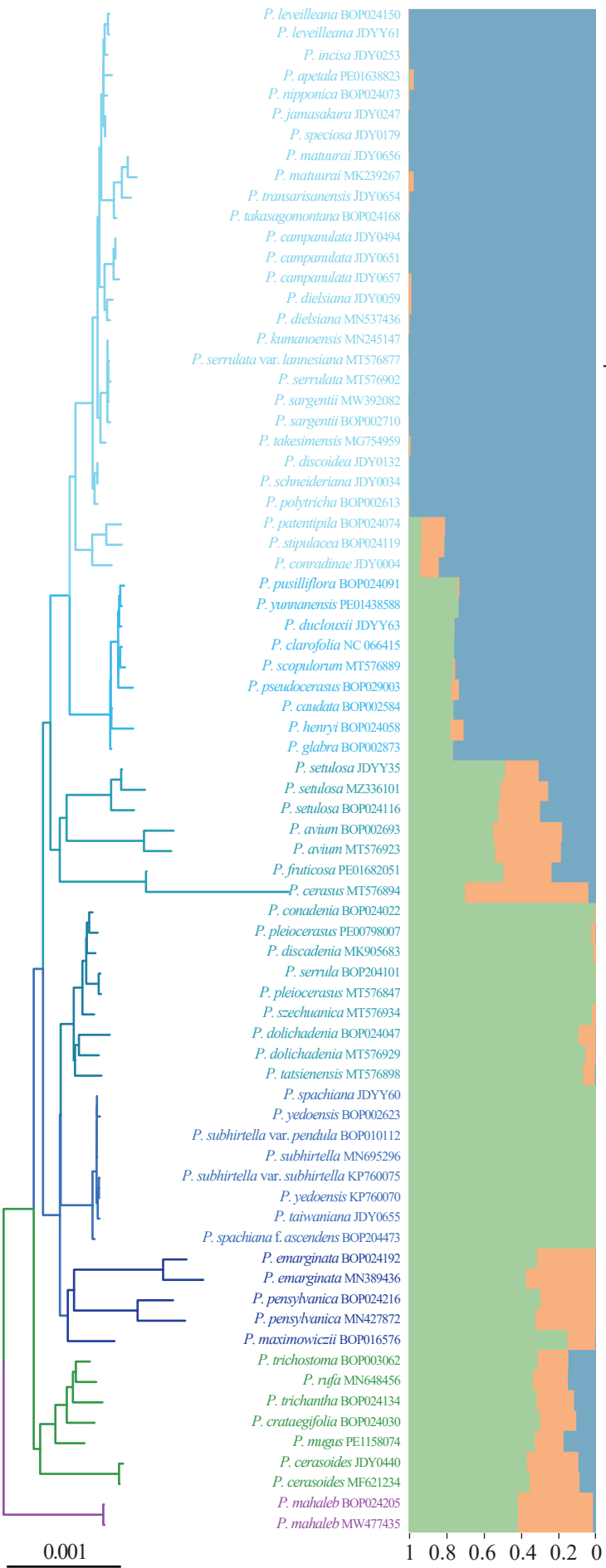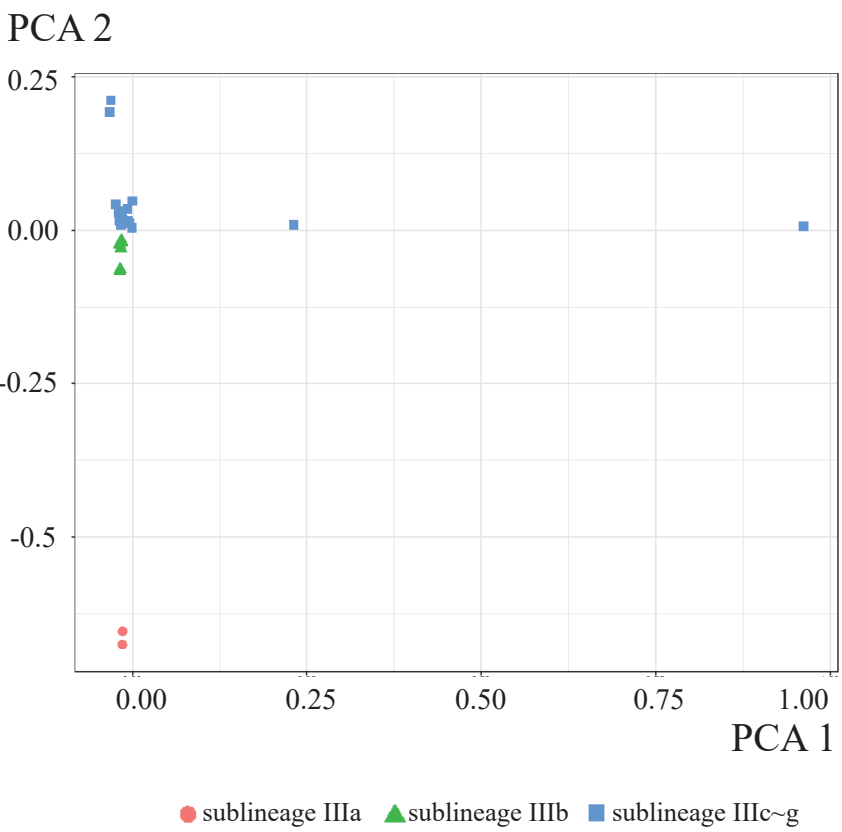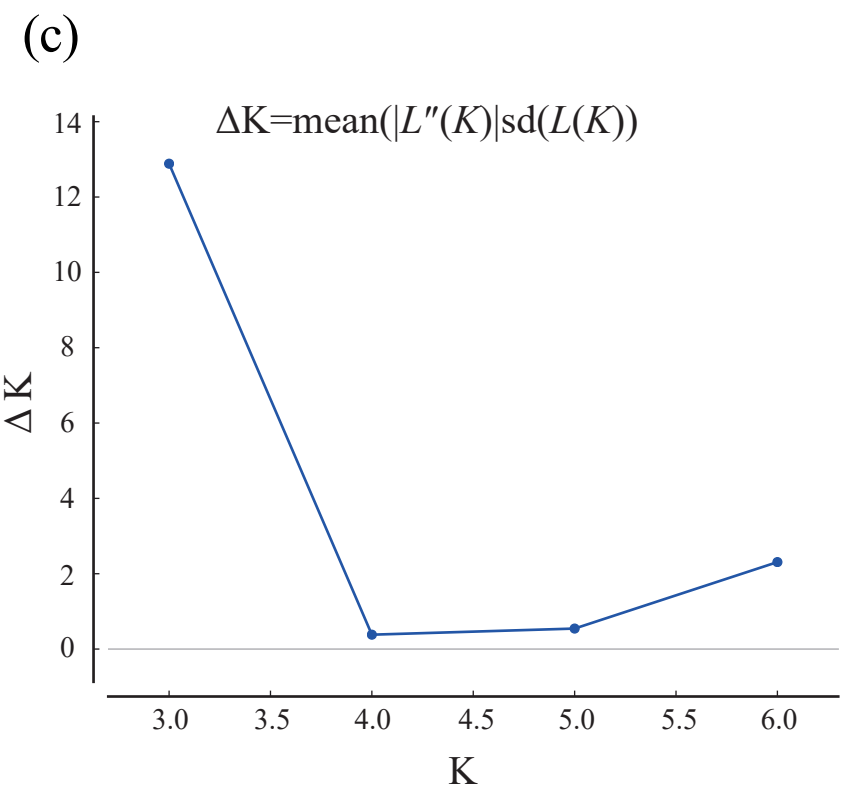

Supplement: Supplementary file 1 [file ijms-24-15612-s001.zip › Figure S5.pdf]
